# GLP-1 Agonism for Kidney Transplant Recipients: A Narrative Review of Current Evidence and Future Directions Across the Research Spectrum

**DOI:** 10.1177/20543581241290317

**Published:** 2024-10-31

**Authors:** Victoria J. Riehl-Tonn, Kyle D. Medak, Christie Rampersad, Anne MacPhee, Tyrone G. Harrison

**Affiliations:** 1 Department of Medicine, University of Calgary, AB, Canada; 2 Libin Cardiovascular Institute, Cumming School of Medicine, University of Calgary, AB, Canada; 3 Lunenfeld-Tanenbaum Research Institute, Sinai Health, Toronto, ON, Canada; 4 Ajmera Transplant Centre, Toronto General Hospital, University Health Network, ON, Canada; 5 Institute of Health Policy, Management and Evaluation, University of Toronto, ON, Canada; 6 Canadians Seeking Solutions and Innovations to Overcome Chronic Kidney Disease (Can-SOLVE CKD), Vancouver, BC, Canada; 7 Department of Community Health Sciences, University of Calgary, AB, Canada; 8 O’Brien Institute for Public Health, Cumming School of Medicine, University of Calgary, AB, Canada

**Keywords:** kidney transplant, diabetes, kidney disease, obesity, cardiovascular outcomes

## Abstract

**Purpose of Review:**

Diabetes is the most common cause of kidney disease in individuals that receive a kidney transplant, and those without pre-existing diabetes are at greater risk of developing diabetes following kidney transplant. A class of diabetes treatment medications called glucagon-like peptide-1 receptor agonists (GLP-1RA) has seen recent widespread use for people with diabetes or obesity, with efficacy for improved glycemic control, weight loss, and reduced risk of cardiovascular events. Given these benefits, and indications for use that often co-occur in kidney transplant recipients, use of GLP-1RAs warrants consideration in this population. Therefore, we sought to review the current literature to better understand the mechanisms of action, clinical application, and person-centred considerations of GLP-1RAs in kidney transplant recipients.

**Sources of Information:**

Original articles were identified between December 2023 and July 2024 from electronic databases including the Ovid MEDLINE database, PubMed, and Google Scholar using terms “kidney transplant,” “GLP-1,” “glucagon-like peptide-1 receptor agonist,” and “diabetes.”

**Methods:**

A comprehensive review of the literature was conducted to explore the relationship between GLP-1RAs and kidney transplant recipients. We reviewed the current state of evidence across the research disciplines of basic or fundamental science, clinical and health services research, and person-centred equity science, and highlighted important knowledge gaps that offer opportunities for future research.

**Key Findings:**

Numerous clinical studies have demonstrated the benefit of GLP-1RAs in people with and without diabetic kidney disease, including decreased risk of cardiovascular events. However, there is a paucity of high-quality randomized controlled trials and observational studies analyzing use of GLP-1RAs in kidney transplant recipients. Evidence of benefit in this population is therefore limited to small studies or inferred from research conducted in nontransplant populations. Growing evidence from preclinical and clinical studies may elucidate renoprotective mechanisms of GLP-1RAs and remove barriers to application of these drugs in the transplant recipient population. Individuals who are female, non-white, have lower socioeconomic status, and live in rural communities are at greater risk of diabetes and have lower uptake of GLP-1RAs. There is a need for clinical trials across diverse kidney transplant populations to estimate the efficacy of GLP-1RAs on important health outcomes.

**Limitations:**

The search strategy for this narrative review may not have been sensitive to identify all relevant articles. Our search was limited to English language articles.

## Abrégé

**Contexte motivant la revue:**

Le diabète est la cause la plus fréquente de maladie rénale chez les personnes qui reçoivent une greffe de rein, et celles qui ne souffrent pas d’un diabète préexistant courent un plus grand risque de développer la maladie après la greffe. Les agonistes des récepteurs du peptide-1 de type glucagon (GLP-1RA), une classe de médicaments pour le traitement du diabète, ont récemment fait l’objet d’une utilisation répandue chez les personnes obèses ou atteintes de diabète en raison de leur efficacité dans le contrôle glycémique, la perte de poids et la réduction du risque d’événements cardiovasculaires. Compte tenu de ces bienfaits et des indications d’utilisation qui coïncident chez les receveurs d’une greffe rénale, l’utilisation des GLP-1RA mérite d’être envisagée dans cette population. Nous avons examiné la documentation actuelle afin de mieux comprendre les mécanismes d’action, les applications cliniques et les aspects centrés sur la personne des GLP-1RA chez les receveurs d’une greffe rénale.

**Sources de l’information:**

Les articles originaux ont été répertoriés entre décembre 2023 et février 2024 dans les bases de données électroniques, notamment Ovid MEDLINE, PubMed et Google Scholar, en utilisant les termes anglais « kidney transplant », « GLP-1 », « glucagon-like peptide-1 receptor agonist » et « diabetes ».

**Méthodologie:**

Un examen approfondi de la documentation a été réalisé afin d’explorer la relation entre les GLP-1RA et les receveurs d’une greffe rénale. Nous avons examiné l’état actuel des données probantes dans les disciplines de recherche de la science fondamentale, de la recherche clinique et des services de santé, ainsi que de la science de l’équité centrée sur la personne. Nous avons mis en évidence d’importantes lacunes dans les connaissances offrant des possibilités de recherches futures.

**Principaux résultats:**

Plusieurs études cliniques ont démontré les bienfaits des GLP-1RA chez les personnes atteintes ou non de maladie rénale diabétique, notamment une réduction du risque d’événements cardiovasculaires. Il existe cependant peu d’essais contrôlés randomisés et d’études observationnelles de haute qualité ayant examiné l’utilisation des GLP-1RA chez les receveurs d’une greffe rénale. Les preuves de bénéfices pour cette population sont limitées à de petites études ou déduites de recherches menées dans des populations de personnes non transplantées. Un nombre croissant de données issues d’études précliniques et cliniques pourrait aider à expliquer les mécanismes rénoprotecteurs des GLP-1RA et à éliminer les obstacles à l’utilisation de ces médicaments dans la population des receveurs de greffe. Les personnes de sexe féminin, non blanches, de statut socio-économique plus faible et résidant dans des communautés rurales présentent un risque plus élevé de développer le diabète et une plus faible adhérence aux GLP-1RA. Il est nécessaire de mener des essais cliniques auprès de diverses populations de greffés du rein afin de pouvoir estimer l’efficacité des GLP-1RA sur d’importants résultats de santé.

**Limites:**

La stratégie de recherche employée pour cette revue narrative pourrait ne pas être assez sensible et avoir manqué des articles pertinents. Notre recherche était limitée aux articles rédigés en anglais.

## Introduction

Kidney disease occurs in up to 40% of individuals living with diabetes, making diabetes the primary cause of kidney failure (KF) worldwide.^1,2^ Many affected individuals require kidney replacement therapy with maintenance dialysis or kidney transplantation. Despite clinical advancements, mortality rates remain higher in people with diabetes after kidney transplantation compared with those without,^3^ emphasizing the importance of treatments and prevention of diabetes to improve outcomes in individuals living with KF. In kidney transplant recipients, 38% have diabetes pre-transplant,^4^ and 4% to 25% develop post-transplant diabetes mellitus (PTDM).^5^ Transplant-specific risk factors for PTDM include increased glucocorticoids, infection, higher human leukocyte antigen mismatches, and rejection,^6^ highlighting the need for interventions targeting both kidney disease and diabetes.

The complications of type 2 diabetes (T2D) are systemic, including macrovascular (ischemic heart disease, stroke, peripheral vascular disease) and microvascular complications (nephropathy).^7^ In kidney transplant recipients, diabetes may lead to diabetic kidney disease in the allograft, cardiovascular (CV) disease, infections, and mortality.^8,9^ Unfortunately, diabetes treatment options are limited in kidney transplant recipients where medication interactions pose potential risks. High-quality evidence in these populations is often lacking, leading clinicians to attempt to extrapolate from non-kidney transplant populations. Despite significant growth of diabetes treatment options in recent years for people with and without kidney disease, guideline-directed therapies for transplant patients remain limited.

Glucagon-like peptide-1 receptor agonists (GLP-1RAs) are one such class of antihyperglycemic that has demonstrated efficacy for people with diabetes across the spectrum of CV^10^ and kidney disease outcomes.^11,12^ Despite evidence that GLP-1RAs may be beneficial in managing chronic kidney disease (CKD) in people living with diabetes,^12^ they are largely unaccounted for in contemporary guidelines for kidney transplant recipients. The 2020 Kidney Disease: Improving Global Outcomes (KDIGO) Clinical Practice Guideline for Diabetes Management in CKD^13^ recommended first-line use of metformin for kidney transplant recipients with T2D and estimated glomerular filtration rate (eGFR) ≥30 mL/min/1.73m^2^, noting that future studies were needed to confirm safety and clinical benefit of GLP-1RAs. In this narrative review of evidence across research disciplines, we aim to summarize the current mechanistic and clinical evidence, discuss social considerations, and highlight future directions for GLP-1RAs use in kidney transplant recipients.

## Methods

Original articles and relevant reviews were identified from Ovid MEDLINE database, PubMed, and Google Scholar, using search terms including and related to “kidney transplant,” “GLP-1,” “glucagon-like peptide-1 receptor agonist,” and “diabetes.” Our search focused on the major research disciplines of basic or fundamental science, clinical and health services research, and equity, diversity, inclusion, and access (EDIA) principles. Where no relevant studies were identified in the kidney transplant population, we extended our search iteratively to KF, CKD, and the general population. Our searches were conducted between December 2023 and July 2024, and limited to English language articles. Our review follows this overall structure, reporting findings in kidney transplant recipients followed by KF, CKD, and the general population, as we summarize the current state of the evidence.

## Mechanism of Action for GLP-1 and GLP-1RAs to Induce Improvements in the Kidney

Gluacgon-like peptide-1 (GLP-1) is an incretin hormone released by enteroendocrine cells of the gut. Release of GLP-1 following a meal supports an insulin response, mitigating a rise in blood glucose.^14^ Beyond the stimulation of glucose-dependent insulin secretion from the pancreas, GLP-1 also controls glycemia by inhibiting food intake, glucagon secretion, gastric emptying, and possesses protective actions in pancreatic beta cells including survival, proliferation, and biosynthesis of the insulin precursor, proinsulin.^14^ GLP-1 receptors (GLP-1R) are additionally found in other organs such as the brain, lungs, gut, and kidneys.^15^ These actions make GLP-1R an effective target for T2D therapeutics to potentially mitigate systemic effects of diabetes.

Although endogenous GLP-1 is short-lived (half-life of ~2 minutes), long-lasting GLP-1RAs have half-lives of hours to days^16^ and maintain their efficacy after repeated pharmacological doses.^17^ GLP-1RA treatments have shown to reduce albumin excretion in people with T2D,^18^ in addition to enhancement of diuresis and natriuresis.^19^ Although the canonical GLP-1R is expressed in the kidney, attempts to uncover its localized action have been challenging. The literature describing GLP-1R expression in the kidney is rife with misattribution of cell types expressing GLP-1R due to extensive use of nonvalidated GLP-1R antibodies.^20,21^ Very low expression of GLP-1R mRNA challenges detection sensitivity when assessing transcription in individual cells.^22^ Nevertheless, there is reasonable agreement^23-25^ that GLP-1R in the kidney is predominantly located in subsets of vascular smooth muscle cells, as shown in mice, monkeys, and humans.^22,25,26^ When these tissues are studied ex vivo, the kidney vascular smooth muscle GLP-1Rs are important for blood flow control to the kidney.^26^ How these functions improve kidney outcomes is not fully elucidated; however, preclinical data in mice and other models demonstrate that GLP-1R loss exacerbates kidney injury, whereas its activation is protective of kidney function.^27,28^ Outside of vascular smooth muscle, the mechanisms by which GLP-1 action can reduce kidney injury are likely multifactorial,^29,30^ reflecting that multiple tissues are contributing to improvements. There is growing recognition of increased CKD rates in people with obesity without T2D.^31^ Mechanistically, an obesity-inflammation axis can drive CKD^31^ and recent evidence in mouse models show brain GLP-1R, which is enriched in the hypothalamus and brainstem^32^ (critical areas for immune response regulation),^33^ may produce anti-inflammatory effects systemically.^34^ Research in rodents and humans show that GLP-1RAs improve circulating glucose, blood pressure, inflammation, and dyslipidemia both independent of, and secondary to, weight loss.^29,35,36^ GLP-1RA improves kidney blood flow while reducing kidney and systemic inflammation, albuminuria, harmful reactive oxygen species formation, and kidney fibrosis in preclinical rodent models.^26,29-31,34^ Simultaneously, these improvements are mirrored in reduced rates of CKD progression in people with T2D or obesity treated with GLP-1RAs.^34,35,37,38^ Other incretin therapies, such as activation of the gastric inhibitory polypeptide receptor (GIPR), have not been thoroughly studied in the kidney, consistent with a lack of detectable kidney GIPR expression^39^; thus, we have not focused our literature summary on this topic. Much excitement surrounds molecules which target multiple incretin receptors simultaneously, such as GLP-1-GIP co-agonists and molecules which both agonize the GLP-1R and antagonize the GIPR. Impressively, both of these approaches have achieved greater weight loss than targeting each receptor alone,^40-42^ though there is ambiguity in understanding how this works and any direct effects on the kidney likely rely on GLP-1 signaling.^25,26^

In kidney transplant recipients, calcineurin inhibitors or mammalian target of rapamycin inhibitors are commonly prescribed and result in pancreatic beta cell dysfunction while GLP-1RAs completely or partially recover these cells.^43^ Although calcineurin inhibitors significantly increase the risk of PTDM, they remain standard of care in this population^44^ to optimize graft survival. The GLP-1RAs may play a role in preventing and managing PTDM through glycemic control and weight loss.^45^ While animal studies suggest that GLP-1RAs have local functions on the pancreas,^46-49^ the systemic effects to prevent or ameliorate PTDM are worthwhile. Many of the aforementioned mechanisms apply to kidney transplant populations but are confounded by additional hemodynamic or vascular-mediated risk factors for allograft dysfunction and exacerbated by post-transplant immunosuppression.^50^ Human studies to support these claims are lacking and limited to small case series.^51^

## Clinical, Health Services, and Population-Based Research

**Figure 1.**
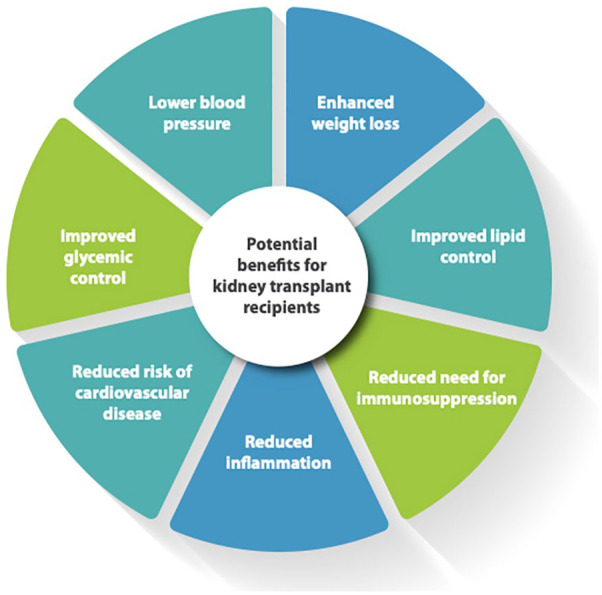
Potential benefits and mechanisms of action of GLP-1RAs for kidney transplant recipients.

Thus far, we have primarily reviewed fundamental and translational science on GLP-1RAs as it applies to kidney health (Figure 1). Clinical trial settings are ideal for examining the efficacy of novel therapies or therapies in novel populations, given the strict study conditions and benefits of randomization. However, evaluation of whether these findings generalize to transplant and CKD populations when not under clinical trial settings is challenging, necessitating health services and population-based epidemiologic studies. We summarize these research domains here, with application to kidney transplant populations where available and appropriate.

### Observational and Population-Based Research in Kidney Transplant Recipients

The GLP-1RA’s impact on long-term health outcomes for people with CKD has only recently been a research focus. As GLP-1RAs are increasingly studied in kidney disease settings and receive approval for clinical use beyond diabetes and obesity, we will be able to examine outcomes in people with kidney disease at a population level. Given the paucity of health services research on GLP-1RA in the kidney transplant and CKD populations, we are left extrapolating suspected impacts modeled in other populations. Reports of GLP-1RA in kidney transplant recipients are limited to small observational studies where evidence-based benefits in the general population have not been convincingly demonstrated. These real-world accounts provide important information on safety and implementation in clinical practice which may be leveraged for future trials; this evidence is summarized below and in Table 1.

**Table 1.** GLP-1RAs and Kidney Outcomes: An Overview of Important Trials, Observational Studies, and Meta-analyses.

Study	Study design	Treatment	Health status	Sample size	Kidney outcomes (primary vs secondary)	Results
**Kidney transplant recipients**						
HALLMARK (recruitment in progress)^52^	RCT	Combination Semaglutide and Dapagliflozin	With and without T2D	20	GFR, eGFR, urinary albumin excretion, incidence of AKI (Secondary)	Study ongoing
Krisanapan et al^53^	Meta-analysis	GLP-1RAs		338	eGFR, serum creatinine, UPCR (Primary)	• No change in eGFR (SMD −0.07 mL/min/1.73m^2^, 95% CI −0.64, 0.50) or creatinine [SMD −0.08 mg/dL, 95% CI −0.44, 0.28). • Significant decrease in UPCR (SMD -0.47, 95% CI −0.77 to −0.18).
Mahmoud et al^54^	Retrospective Study	GLP-1RAs and SGLT2i	T2D Baseline eGFR ≥ 25 mL/min	GLP-1RA (n=41), SGLT2i (n=98), control (n=70)	eGFR, UACR (Primary)	• eGFR in SGLT2i and GLP-1RA not significantly different compared with control. • Sub-analysis by CKD stage showed improvement in eGFR ≥90 with SGLT2i, dip eGFR in SGLT2i at 1-3 months. Reduction in albuminuria in SLGT2i and GLP-1RA
Sato et al^55^	Retrospective Study	GLP-1RAs	T2D At least 2 years follow-up post-transplant	73 recipients on GLP-1RAs, 73 recipients not using GLP-1RAs	eGFR (sustained reduction of at least 40% from baseline for 4 months post-transplant) (Primary)	• 40% sustained eGFR reduction for 4 months post-transplant with GLP-1RAs (OR 0.105; 95% CI 0.012, 0.961; *p* = .046), • Recipients with sustained eGFR reduction of >40% for 4 months experienced graft loss.
Vigara et al^56^	Retrospective Study	GLP-1RAs	T2D	40 (follow-up of 6 months) and 26 (follow-up of 12 months)	eGFR, UACR (Primary)	• Improved eGFR by +3.5 mL/min/1.73m^2^ at 12 months (*p* = .03) • Reduced proteinuria of -59.1 mg/g at 6 months (*P* = .009) and -48.5 mg/g at 12 months (*P* = .021)
Sweiss et al^57^	Retrospective Study	GLP-1RA with diabetes	SOT	118	Serum creatinine and eGFR (Secondary)	Significant change in serum creatinine (median change -0.0 [IQR -0.01, -0.23], *P* < .0001) and eGFR (median change 5 [IQR 0, 13], *P* < .0001)
Yugueros González^58^	Retrospective Study	GLP-1 analogues and/or SGLT2	With and without T2D	Diabetes (n=10), Obesity without Diabetes (n=5)	Serum creatinine and 24-hour proteinuria (Secondary)	No significant change in serum creatinine or proteinuria
Kukla et al^59^	Retrospective Study	GLP-1RAs	T2D	17	Change in serum creatinine and eGFR (Secondary)	eGFR and creatinine remained stable
Thangavelu et al^60^	Retrospective Study	GLP-1RA	SOT	19	eGFR (Unclear)	No changes in eGFR
Singh et al^61^	Retrospective Study	Dulaglutide and Liraglutide	SOT	Dulaglutide (n=63), Liraglutide (n=25)	All-cause graft failure (Primary), serum creatinine, and eGFR (Secondary)	• No difference in graft survival • 10% reduction in creatinine and 15% reduction in eGFR with dulaglutide • 7% increase in creatinine and 8% increase in eGFR with liraglutide
Singh et al^62^	Retrospective Study	Dulaglutide	SOT with diabetes	63	Serum creatinine, eGFR, all-cause graft failure (Secondary)	• No difference in creatinine or eGFR • No increased risk in graft failure
Liou et al^63^	Retrospective Study	Liraglutide	T2D	7	Serum creatinine, eGFR, UPCR (Secondary)	• Improved eGFR from initial 67.7 ± 18.7 to a nadir of 76.5 ± 18.7 mg/dL. • No significant change in UPCR
**Chronic kidney disease**						
FLOW^12^	RCT	Semaglutide	T2D	Semaglutide (N=1767), Placebo (N=1766)	Major kidney disease event (initiating KRT, death from kidney causes, persistent reduction in eGFR to <15 mL/min/1.73m^2^ sustained for ≥28 days, KF) (Primary)	24% reduction in major kidney disease event
REMODEL (upcoming)^64^	RCT	Semaglutide	T2D	Estimated 105	Change in kidney oxygenation, global kidney perfusion, and kidney inflammation (Primary); change in gene expression, glomerular basement membrane width, apparent diffusion coefficient, mean renal artery resistive index, mean arterial flow, natriuresis, albumin excretion, and creatinine clearance (Secondary)	Recruitment ongoing, awaiting results
OK-TRANSPLANT 2 (upcoming)^65^	RCT	Semaglutide	Obesity, high-risk CKD/dialysis that are kidney transplant candidates	60	% Change in HbA_1c_, change in 2-week fasting glucose, change in 2-week glycemic variability and time in rage in order to be eligible for kidney transplant	Not yet Recruiting, doing feasibility trail initially
**Cardiovascular outcomes**						
Kristensen et al^66^	Meta-analysis	GLP-1RAs	Individuals with T2D	56,004	Development of new-onset macroalbuminuria, 40% decline in eGFR, doubling of serum creatinine, initiating KRT, death from kidney cause (Primary)	17% reduction in the risk of worsening kidney outcomes
LEADER^10^	RCT	Liraglutide	Individuals with T2D	Liraglutide (N=4668), placebo (N=4672)	Diabetic nephropathy (Secondary)	Lower rates of nephropathy (1.5 vs 1.9 events per 100-patient years in liraglutide vs placebo (HR 0.78, 95% CI: 0.67,0.92)
SUSTAIN^67^	RCT	Semaglutide	Individuals with T2D	Semaglutide (n=1648), placebo (n=1649)	Creatinine clearance, serum creatinine, macroalbuminuria, or KRT (Secondary)	Reduced risk of new or worsening nephropathy (3.8% in semaglutide and 6.1% in placebo) (HR 0.64, 95% CI: 0.46, 0.88)
REWIND^68^	RCT	Dulaglutide	Individuals with T2D with previous CV event or CV risk factors	Dulaglutide (n=4949), placebo (n=4952)	UACR >300 mg/g, sustained >30% eGFR decline, KRT (Secondary)	• Reduced rate of macroalbuminuria, KRT, and sustained eGFR decline of 30%
SELECT^37^ and post hoc analysis^69^	RCT	Semaglutide	Obese individuals (<45 years of age) with CVD	Semaglutide (n=8803), placebo (n=8803)	Composite kidney end point including death from kidney disease, initiation of chronic KRT, onset of persistent eGFR < 15 mL min^−1^ 1.73 m^−2^, persistent ≥50% reduction in eGFR or onset of persistent macroalbuminuria. Compared change in eGFR over 2 years. (Primary)	• 22% lower risk of composite kidney outcome • Slower eGFR decline at 2 years for all treated with semaglutide (0.75 mL/min/1.73m^2^ difference) or for people with eGFR <60 mL/min/1.73m^2^ at baseline (2.19 mL/min/1.73m^2^ difference).
STEP 1-3 (Post hoc analysis)^11^	RCT, post hoc analysis	Semaglutide	Obese individuals (STEP 1 and 3) and with T2D (STEP 2)	Semaglutide 1.0 mg (n=403), Semaglutide 2.4 mg (n=404), placebo (n=403)	UACR (STEP2), changes in eGFR (STEP1-3 pooled) (Post hoc analysis)	• 14.8% reduction in UACR in semaglutide 1.0 mg • 20.6% reduction in UACR in semaglutide 2.4 mg • No change in eGFR
SURPASS-4^70^	RCT, post hoc analysis	Tirzepatide	T2D	Tirzepatide (n=995), insulin glargine (n=1000)	eGFR, Composite kidney endpoint (new-onset macroalbuminuria, at least 40% eGFR decline, ESKD, death from KF)	• Tirzepatide slowed rate of eGFR decline compared with insulin glargine (between-group difference: 2.2, 95% CI: 1.6, 2.8) • 42% reduced the risk of composite kidney endpoint (HR 0.58, 95% CI: 0.32, 0.80)

*Note.* AKI = acute kidney injury; CV = cardiovascular; eGFR = estimated glomerular filtration rate; ESKD = end-stage kidney disease; FLOW = effect of semaglutide versus placebo on the progression of renal impairment in subjects with type 2 diabetes and chronic kidney disease; GFR = glomerular filtration rate; GLP-1RA = Glucagon-like peptide-1 receptor agonist; HALLMARK = The efficacy, mechanism and safety of sodium-glucose co transporter 2 inhibitor and glucagon-like peptide-1 receptor agonist combination therapy in kidney transplant recipients; KF = kidney failure; KRT = kidney replacement therapy; LEADER = liraglutide effect and action in diabetes evaluation of cardiovascular outcome results; mGFR = measured glomerular filtration rate; REMODEL = Renal Mode of Action of Semaglutide in Patients With Type 2 Diabetes and Chronic Kidney Disease; REWIND = dulaglutide and cardiovascular outcomes in type 2 diabetes; SELECT = semaglutide and cardiovascular outcomes in obesity without diabetes; SGLT2i = Sodium-glucose cotransport 2 inhibitors; SOT = solid organ transplant; STEP1-3 = semaglutide treatment effect in people with obesity; SUSTAIN = trail to evaluate cardiovascular and other long-term outcomes with semaglutide in subjects with type 2 diabetes; T2D = type 2 diabetes; UACR = urine albumin to creatinine ratio; UPCR = urine protein to creatinine ratio.

#### Weight loss, cardiovascular disease, and metabolic syndrome

Although GLP-1RAs are Health Canada and FDA-approved for chronic weight loss, retrospective cohorts of kidney transplant recipients experienced no or variable weight loss of 0.5-9.9 kg, with slightly greater weight loss observed with dulaglutide versus liraglutide.^56-63,71-73^ However, these studies had variable follow-up periods (~6-24 months) and baseline weight. Studies reporting body mass index (BMI) showed moderate reductions of 1.63-2.01 kg/m^2^ after GLP-1RAs initiation.^60,62^ Mahmoud et al^54^ described 0.34 kg/m^2^ lower BMI in recipients receiving GLP-1RAs, versus 0.015 kg/m^2^ gain in controls. Another retrospective study on kidney transplant recipients (>14 years of age) with pre-existing T2D and PTDM found semaglutide reduced HbA_1c_ and weight.^45^ OK-TRANSPLANT 2 is an upcoming randomized controlled trial (RCT) in potential kidney transplant recipients experiencing obesity, and will examine the effect of semaglutide on diabetes and weight loss.^65^

CV disease is the leading cause of mortality in CKD,^74^ including kidney transplant recipients,^75^ and is particularly elevated in those with diabetes.^76^ However, there are no studies on GLP-1RAs and CV outcomes in kidney transplant recipients, highlighting a key area for future research.^77^ In addition, following potential benefits in metabolic syndrome and trials demonstrating improved liver fibrosis and fat deposition in the general population, a single-centre retrospective study of 29 liver transplant recipients found GLP-1RAs were associated with weight loss and improved glycemic control.^78-81^ Although none of these patients had combined kidney transplant, these findings may be of interest to multi-organ kidney transplants and additional subpopulation studies are needed.

#### Glycemic control and PTDM

In people with diabetes, clinical efficacy of GLP-1RAs for glycemic control has varied in kidney transplant cohorts, with HbA_1c_ reductions of 0.5% to 2% compared with baseline.^57-60,62,63^ A recent retrospective study by Mahmoud et al^54^ compared 41 kidney transplant recipients on GLP-1RAs with 70 patients on standard of care medications for diabetes. Recipients on GLP-1RAs experienced 0.4% reduction in HbA_1c_ after 1 year, while there was no change in the control group (*P* = .009).^54^ Indirect support for antihyperglycemic efficacy is provided by required dose reduction of other antihyperglycemic agents after GLP-1RA initiation, to avoid hypoglycemia. Total daily insulin dose reduction ranged from 4 to 29 units across observational cohorts of transplant recipients after initiating GLP-1RAs.^59^ A larger insulin dose reduction was observed with dulaglutide versus liraglutide (26% vs 3.6%, *P* = .01) in a single-centre cohort of 88 solid organ transplant recipients that included 88% kidney transplant recipients.^61^

#### Kidney function

A recent meta-analysis by Krisanapan et al^53^ included 9 cohort studies (no relevant trials identified) with a pooled total of 338 kidney transplant recipients; they found GLP-1RAs did not significantly change eGFR (standard mean difference [SMD] −0.07 mL/min/1.73m^2^; 95% confidence interval [CI] −0.64 to 0.50) or creatinine (SMD −0.08 mg/dL; 95% CI −0.44 to 0.28) (Table 1). However, they found a significant reduction in HbA_1c_ (SMD −0.85%, 95% CI: −1.41, −0.280) and urine protein to creatinine ratio (SMD −0.47; 95% CI −0.77, −0.18). Several retrospective kidney transplant cohort studies have described stable or modestly improved eGFR from baseline (3.5-5 mL/min/1.73m^2^) with up to 2 years of follow-up.^54,57,59,60,62,63^ The GLP-1RA use was associated with lower risk of at least 40% sustained eGFR reduction at 4 months post-transplant in a retrospective cohort of Japanese kidney transplant recipients with propensity score matching of 73 patients on GLP-1RAs and 73 patients with non-GLP-1RAs antihyperglycemic medications (odds ratio 0.105, 95% CI: 0.012, 0.961; *P* = .046).^55^ Interestingly, Singh et al^61^ described 15% increased eGFR compared with 8% reduced eGFR from baseline in kidney transplant recipients treated with dulaglutide and liraglutide, respectively. Conceivably, differential effects of GLP-1RAs agents within the drug class may have contributed to lack of clinical efficacy in studies where these agents were pooled together in analysis. The GLP-1RAs have also been associated with minimal or no proteinuria reduction in small studies including kidney transplant recipients.^54,56,58,59^ Studies with longer follow-up could potentially explore whether this risk factor modification may translate into improved graft survival.

#### Allograft failure and mortality

Two observational retrospective studies with duplicate cohorts reported no difference in all-cause graft failure, defined as a functioning graft with no requirement for dialysis or relisting for transplant, at up to 2 years follow-up.^61,62^ Despite evidence for improved kidney function with GLP-1RAs,^56,57,63^ larger studies with longer-term follow-up and control groups are needed to ascertain whether GLP-1RAs may modify hard outcomes of allograft survival. We did not identify any studies on allograft rejection or mortality risk.

#### Drug dosing and drug-drug interactions

Delayed gastric emptying with GLP-1RA may impact oral medication absorption, including immunosuppressive agents. There are few GLP-1RA drug interactions and dose adjustments are not necessary, though, given the risk of gastrointestinal (GI) effects, these medications are started at low doses and titrated slowly. Dose adjustments may be warranted depending on the intended use of antihyperglycemic medications (eg, weight loss or diabetes management), but also vary in CKD or kidney transplant recipients depending on the medication and eGFR.^82^ However, due to minimal studies of GLP-1RA in kidney transplant, we are unaware of how dose adjustments may differ if based on eGFR values in this population. It is important to consider dose adjustment to mitigate hypoglycemia when other antihyperglycemic agents are used in combination with GLP-1RAs.^59,61^

### Clinical Use of GLP-1RA in Non-Kidney Transplant Populations: From Diabetes to Other Contexts

Over the past decade, RCTs have extended the roles of popular glucose-lowering drugs, primarily, GLP-1RAs and sodium-glucose cotransport 2 inhibitors (SGLT2is), to prevent complications including CV disease and nephropathy in individuals with T2D.^29^ The open-label pilot HALLMARK study is investigating the mechanisms and safety of combination semaglutide and dapagliflozin, and is currently recruiting kidney transplant recipients with and without T2D.^52^ The trial aims to enroll 20 participants to analyze proximal tubular natriuresis with combination therapy and monotherapy as primary outcomes, and measured glomerular filtration rate, eGFR, and urinary albumin excretion as secondary outcomes.

Other clinical studies such as LEADER (Liraglutide Effect and Action in Diabetes: Evaluation of Cardiovascular Outcome Results)^10^ and REWIND (Researching Cardiovascular Events with a Weekly Incretin in Diabetes)^68^ trials using GLP-1RAs, such as liraglutide and dulaglutide, respectively, have demonstrated kidney protective actions as secondary outcomes in nontransplant populations (Table 1). The SUSTAIN-6 trail found a reduced risk of new or worsening nephropathy with semaglutide in people living with T2D.^67^ Semaglutide has been examined as treatment for obesity, with and without diabetes, in the Semaglutide Treatment Effect in People with obesity (STEP) program of clinical trials. Across the 4 trials, semaglutide consistently reduced weight in people living with obesity. Heerspink et al^11^ examined the effect of semaglutide on albuminuria and eGFR (a change from baseline to follow-up) in an exploratory analysis from STEP1-3. They found reduced albuminuria by 28% to 38% over 68 weeks of follow-up, however, no difference in eGFR. In addition, STEP1-3 estimated that 59% of decreased albuminuria was independent of changes in HbA_1c_ or body weight. Conversely, in a post hoc analysis of the SURPASS-4 RCT of individuals with T2D, tirzepatide (a combined GIP/GLP-1 receptor agonist) slowed eGFR decline compared with insulin glargine (between-group difference: 2.2, 95% CI: 1.6, 2.8) and had a 42% reduced risk of composite kidney endpoint (new-onset macroalbuminuria, at least 40% eGFR decline, end-stage kidney disease, death from KF) (hazard ratio [HR] 0.58, 95% CI: 0.32, 0.80).^70^ In the SELECT trial, semaglutide was examined in people with obesity, without diabetes, and found reduced major adverse cardiovascular events (MACE) composite outcome by 20% (HR 0.80, 95% CI: 0.72, 0.90).^37^ Impressively, semaglutide also reduced risk of a composite kidney endpoint (death from kidney disease, initiation of kidney replacement therapy, eGFR <15 mL/min/1.73m^2^, or persistent 50% reduction of eGFR or macroalbuminuria) by 22%, as a prespecified secondary outcome.^69^ At 104 weeks post initiation, the benefit of semaglutide in terms of eGFR was net 0.75 mL/min/1.73m^2^ over placebo, and was more significant at 2.19 mL/min/1.73m^2^ for people with baseline eGFR <60 mL/min/1.73m^2^.^69^

In the population-based research setting, Wong et al^83^ applied the eligibility criteria from STEP-1 (ie, adults with obesity) and modeled semaglutide’s potential impact on obesity and CV events if used in eligible people from the United States. An estimated 93 million people in the US experience obesity (38% of the adult population), and when they applied estimates for weight and CV risk reduction from STEP-1, they estimated a 46.1% reduction in obesity (43 million fewer obese people) and 1.5 million CV events prevented over 10 years. These findings are likely relevant in Canada, where there is an estimated obesity prevalence of 26.7%.^84^ Approximately 10% of Canadians have CKD (which often co-occurs and is hastened by obesity)^85^; thus, the impact of GLP-1RAs in Canadians with CKD on their risk of long-term obesity and CV disease outcomes is likely substantial, though presently unstudied.

Notably, research on GLP-1RA use in diabetic kidney disease outcomes, such as progression of CKD or KF, is increasing. A meta-analysis of 7 RCTs that investigated the impact of GLP-1RAs in T2D revealed that this medication class reduced overall risk of MACE by 12% (HR 0.88, 95% CI: 0.82, 0.94).^66^ In addition, GLP-1RAs reduced composite kidney outcome (new-onset macroalbuminuria, decline in eGFR, progression to KF, or death from kidney causes) by 17% (HR 0.83, 95% CI: 0.78, 0.89). In a subgroup of people with eGFR <60 mL/min/1.73m^2^, the risk reduction was similar for MACE though not significant (HR 0.88, 95% CI: 0.76, 1.03).

Similarly, reduced major kidney endpoints in people with diabetes on GLP-1RAs were found in a nationwide Taiwanese cohort of 7279 propensity-matched pairs, with a comparator group taking long-acting insulin.^86^ Chen et al^87^ examined a retrospective cohort of 27 279 nontransplant KF with diabetes on dipeptidyl peptidase-4 inhibitors or GLP-1RAs, and compared their risk of all-cause, sepsis-related or infection-related mortality. They found a 21% lower risk of all-cause mortality (HR 0.79, 95% CI: 0.63, 0.98) and 39% reduction in sepsis-related and infection-related mortality (HR 0.61, 95% CI: 0.40, 0.91) with GLP-1RAs. Plausibly, these results may be extrapolated to kidney transplant recipients with diabetes, and the reduction in infection-related mortality is a finding worth exploring in transplant recipients with active immunosuppression.

To our knowledge, there are no RCTs investigating GLP-1RA use in kidney transplant populations. However, the recently published and landmark FLOW trial is the first RCT dedicated to evaluating the effect of the GLP-1RA semaglutide in people with CKD and T2D with primary kidney outcomes.^88^ The study found that semaglutide reduced the risk of major CKD events, defined as onset of KF (ie, long-term dialysis, kidney transplant, or reduced eGFR to <15 mL/min/1.73m^2^ for ≥28 days), >50% reduction in eGFR from baseline, or kidney or CV-related death.^12^ Another study called REMODEL is underway, which is designed to understand how GLP-1RAs mechanistically work on the kidneys in individuals with CKD and T2D.^64^ Although kidney transplant recipients were excluded from both studies, findings from FLOW and REMODEL are more generalizable to these groups than other GLP-1RA trials.

### Health Economics Considerations

To our knowledge, there are no studies that have examined the economic impact in kidney transplant groups, though with population-based estimates on poor health outcome reduction, the resulting impact on health services use and cost is of additional interest. In Taiwan, investigators used the population-based drug registry and administrative health data sets to model GLP-1RA cost-effectiveness.^89^ They found that GLP-1RAs were cost-effective compared with long-acting insulins, with an incremental cost-effectiveness ratio (ICER) of $6053 USD per quality-adjusted life year (QALY) gained. When estimated for CKD populations, GLP-1RAs were associated with $673 cost savings compared with long-acting insulin and an ICER of $1675 per QALY gained. In sensitivity analyses, the cost of GLP-1RAs was primarily responsible for changes in ICER. With additional population-based data documenting GLP-1RA use and cost in KF and kidney transplant populations, the impacts may be estimated. Furthermore, as the cost of GLP-1RAs may vary within Canada by province and insurance type, these economic impacts must be examined considering the differential cost based on location.^90^

## EDIA for Population Health: Person-Centred Considerations

Factors influencing GLP-1RA use and outcomes including sex/gender, medication burden, race/ethnicity, and socioeconomic status (SES) are of interest. Although obesity, as a risk factor of T2D, is more common in females, males are more often diagnosed with T2D at a younger age and lower BMI,^91^ with male sex also being associated with increased risk of PTDM.^92^ The GI side effects are common with GLP-1RAs (8.5%-23%), including nausea, vomiting, diarrhea, abdominal pain, and decreased appetite,^56,57,59,60,62,63,71^ and primarily occur in females.^93,94^ Mild to severe GI symptoms are common in 20% to 92% of kidney transplant recipients and is often underreported and underestimated by clinicians,^95-99^ however, undoubtedly worsen recipient quality of life.^97,99^ Within a cohort of 17 recipients, GI symptoms were cited as the reason for drug discontinuation in 23%^59^ and may also lead to volume depletion, risk of acute kidney injury, malnutrition, and graft loss.^98^ However, few case reports link exenatide with acute kidney injury, a relationship not observed with other GLP-1RAs^100^ but may result from volume depletion from GI symptoms. Further studies are needed on exenatide as it resists enzymatic degradation requiring elimination by kidney mechanisms.

Gender-related factors may influence symptom reporting as women are believed to be more aware of their health status and more likely to disclose discomfort.^101^ Considering this, accurate prevalence estimates may be difficult to ascertain as minor presentations may be underreported, while more severe presentations precluding drug continuation may not be captured in studies that excluded patients who discontinued the study drug.

Medication burden and nonadherence is common in transplant recipients, with the latter more so among males.^102^ New medications adherence may be further lowered with GLP-1RA initiation in kidney transplant recipients experiencing high medication burden and often taking upward of 20 pills per day.^103-105^ A retrospective study in individuals that discontinued GLP-1RA use, found that 56% of respondents reported injection method of administration as the reason.^106^ However, once-weekly injectable GLP-1RA, semaglutide, increased medication adherence over other injectable administration schedules.^107^ Notably, Kim et al^71^ described a series of 37 kidney transplant recipients where once-weekly dose of dulaglutide alongside basal insulin replaced thrice-daily prandial insulin, with comparable glycemic control. Reduced subcutaneous injections, fewer blood glucose checks, and dose-adjusted insulin at mealtimes may offer improved quality of life and increase adherence compared with traditional diabetes treatment regimens.^71^ Although injection site pain was uncommonly reported,^63,71^ oral formulations of GLP-1RA may still be preferred despite scarce evidence demonstrating CV benefit. A Japanese case report of 3 kidney transplant recipients with metabolic syndrome that safely tolerated oral semaglutide reported that all patients experienced weight loss, 2 had decreased HbA_1c_, and one had decreased albuminuria.^108^

Kidney transplant recipients additionally face cost-related adherence barriers. Although Canadian transplant centres have drug coverage programs for immunosuppressive medications, this may not cover all patients, such as refugees. Drug coverage in US transplant centres is heterogeneous regarding medications and the post-transplant duration covered.^109-111^ Noting insurance coverage variation, kidney transplant recipients may face high direct costs, especially early post-transplant, and medications such as GLP-1RA may generate additional costs.^109-111^ Recent cost estimates for GLP-1RA (~$1200/month) and higher out-of-pocket costs decrease likelihood of initiating GLP-1RA.^112,113^ Such costs may lead to psychosocial stress, worsen socioeconomic risk factors for disease, exacerbate socioeconomic inequities, and increase risk of medication nonadherence.^114^ Within Canada, GLP-1RA availability differs based on provincial insurable drug benefit plan restrictions,^90^ and likely exacerbated by differential cost. In addition, many provincial insurance providers do not match the pace of the advances in GLP-1RA RCT evidence and frequently impose availability restrictions based on several additional lines of therapy prior to arguably more impactful therapies (ie, GLP-1RAs and SGLT2 is inhibitors, etc.)^115-117^

Race, SES, and rural location are also risk factors for T2D^118-120^ and have implications for GLP-1RA use. Black and Hispanic individuals have a higher prevalence of T2D compared with non-Hispanic white individuals after adjusting for age and sex.^119^ Despite increased diabetes risk, a 5-year cohort study found Asian, Black, and Hispanic participants were less likely to receive GLP-1RA treatment^121^ and primarily white patients were using GLP-1RAs or SGLT2is.^122^ However, a systemic review on diabetes medication adherence found Black, Hispanic, and Asian individuals to be more nonadherent compared with white individuals.^123^ Differences in medication adherence may be due to health literacy,^124^ sociocultural background, or differing beliefs and perceptions surrounding treatment.^125^ The GLP-1RA uptake is further decreased by lower annual household income, male sex,^121^ areas of low SES, education, and disadvantaged groups.^126^ In addition, 1 in 10 Canadians receiving a prescription report cost-related nonadherence, which was associated with poor overall health, lower income, and those without drug insurance.^127^ Health care providers cited limited knowledge as the main reason for not prescribing GLP-1RAs, creating an additional barrier.^128^ This may be exacerbated for kidney transplant recipients where primary care providers may not feel comfortable managing this population.^129^

Racial and social disparities also exist within all stages of kidney transplantation. Black race, older age, low income, lack of social support, limited transplant knowledge,^130^ and female sex^131,132^ are associated with lower probability of kidney transplantation. Black patients are less likely to be waitlisted, regardless of age, for kidney transplantation compared with non-Hispanic white patients.^133^ In addition, non-white individuals are at greater risks of PTDM,^134^ including Black^5^ and South Asian^135^ individuals. A prospective cohort study found Black race and experiencing racial discrimination predicted lower adherence,^136^ though not yet found with GLP-1RAs to our knowledge. Another barrier is residence (rural vs urban) as individuals living with CKD in rural communities are at greater risk of mortality and morbidity than those in urban areas.^137,138^ Indigenous individuals living rurally have a 4-fold increased risk of diabetes and 3-fold increased risk of CKD.^139^ Despite these risks, rural location is associated with decreased time to kidney transplantation.^140^ In Australia, a study found that rural or disadvantaged areas are less likely to receive newer diabetes education, including GLP-1RAs and SGLT2is.^141^ To our knowledge, there is no research published regarding geographic location, use, and potential barriers of GLP-1RAs in kidney transplant recipients.

## A Perspective From the Patient-Centred Lens

Our co-author, AM, provided some additional perspective from someone with lived experience of supporting a multi-kidney transplant recipient, with the following commentary:

Diabetes is the most common cause of kidney disease for those who receive kidney transplants. This is a worldwide problem. Research has shown Black and Hispanic people have a higher rate of T2D. Socioeconomic, racial, and social disparities are a barrier for people receiving the proper treatment and medication. Why do we have these disparities and what can be done about it? Cost, education, and affordability are the leading factors driving these disparities. Could a research project be done to include people who are on the fringe and do not get the proper medical attention because of their race, education, and social disparity? If something could be done to reach them, it would be very cost effective. Similar studies have been done with our Indigenous populations and preventive medicine is what is needed.

## Suggestions for Future Research

**Figure 2.**
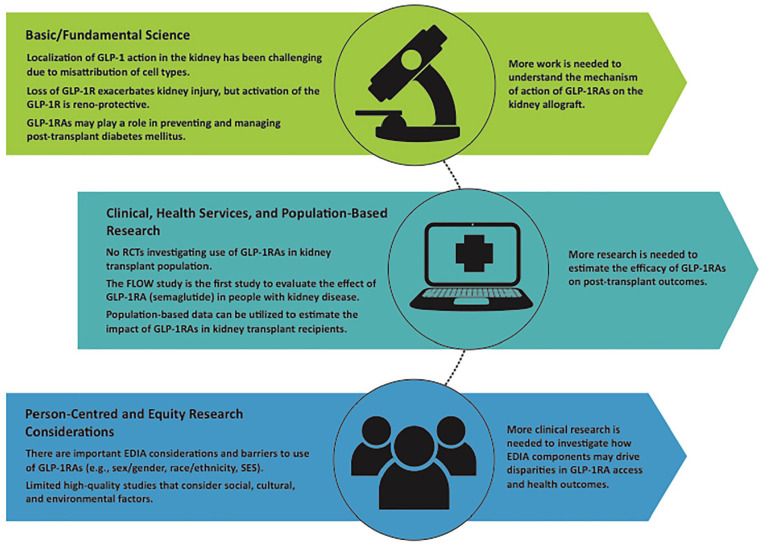
Summary of barriers and knowledge gaps in GLP-1RAs in kidney transplant population across the research disciplines and future directions. *Note.* EDIA = equity, diversity, inclusion, and accessibility; GLP-1 = glucagon-like peptide-1; GLP-1R = glucagon-like peptide 1-receptor; GLP-1RA = glucagon-like peptide-1 receptor agonist; RCTs = randomized controlled trials; SES = socioeconomic status.

Across research disciplines, there are many candidate gaps in the evidence (Figure 2), and with RCTs as the highest level of evidence, this would be an optimal study design to pursue. The findings from the REMODEL study will bridge the gap on how GLP-1RAs mechanistically impact the kidneys. Despite FLOW and REMODEL not including kidney transplant recipients, it will undoubtedly drive interest in preclinical mechanistic studies elucidating the role of GLP-1RAs in the kidney. Although human studies are time-consuming and lack the ability to ascertain the mechanistic underpinning of the metabolic, inflammation, and direct effects of GLP-1RAs, basic studies using genetic rodent models work together with clinical and population-based research. While multiple fields await the important outcomes of future trials performed in kidney transplant recipients, basic research on the complementary contributions of improvements in weight, metabolism, and kidney physiology are yet to be clarified. Concurrently, future clinical evidence is needed to estimate the GLP-1RA efficacy on CV outcomes, mortality, and allograft function. In the population-based research domain, given the widespread use of these agents to treat obesity, researchers with access to population-based data on prescription dispensing can then estimate the impact of GLP-1RAs on kidney outcomes and in kidney transplant recipients on a population level for people without diabetes. Furthermore, as new trial evidence becomes available, we will be able to model the transferred impact of GLP-1RAs on a population level for people with diabetes, allowing anticipated changes to people developing KF, and moreover, those requiring kidney transplantation. While some research has been conducted to understand the important components of EDIA with GLP-1RA use, there is a clear need for further investigation. There is a lack of high-quality studies considering important social and cultural factors with the use of GLP-1RAs in kidney transplant recipients. More clinical research is needed to investigate factors influencing or driving these disparities and barriers to access. As more research is conducted, implementing the findings of GLP-1RA use in kidney transplant recipients will bridge the gap between all areas of research and thus engagement of experts in implementation science is paramount.

## Limitations

This is a narrative review thus relevant articles may not have been captured from our search strategy. In addition, no formal guidelines (eg, Grading and Recommendations, Assessment, Development, and Evaluations [GRADE]) were used. Our search was limited to English language articles.

## Conclusion

Altogether, the available literature suggests that GLP-1RAs are associated with reduced incidence of major kidney disease outcomes, are effective in reducing MACE risk for people with and without diabetic kidney disease and may reduce mortality risk in KF. However, there are limited data examining the role or impact of GLP-1RAs in kidney transplant recipients across all domains of research. Further research is needed to better understand and evaluate the safety and outcomes of using GLP-1RAs in kidney transplant recipients.
